# Psychoeducation and the family burden in schizophrenia: a randomized controlled trial

**DOI:** 10.1186/1744-859X-8-17

**Published:** 2009-07-28

**Authors:** Tanveer Nasr, Rukhsana Kausar

**Affiliations:** 1 Department of Psychology and Applied Psychology, University of the Punjab, Lahore, Pakistan

## Abstract

**Background:**

The majority of patients with schizophrenia live with their relatives in Pakistan, thereby families experience a considerable burden. We aimed to study the impact of psychoeducation on the burden of schizophrenia on the family in a randomised controlled trial.

**Methods:**

A total of 108 patients with schizophrenia and their family members from the outpatient department of a teaching hospital in Lahore, Pakistan were randomised. Both groups received psychotropic drugs but one group received psychoeducation in addition. Family burden was assessed at the time of recruitment and at 6 months post intervention.

**Results:**

In all, 99 patients and their relatives completed the treatment. There was significant reduction in burden at post-intervention assessment in the psychoeducation group based on intention to treat analysis.

**Conclusion:**

Family psychoeducation can be an important intervention for patients with schizophrenia in Pakistan.

## Introduction

There is considerable research evidence on the high levels of financial burden, strain and distress related to caring for an ill family member [1-3]. Families incur costs in terms of psychological strain, social isolation and other practical burdens [4-6]. Emotional strains, financial difficulties and social stigma taken together are referred to as family burden. Hoening and Hamilton [7] attempted to distinguish between objective and subjective burden. The objective burden included the effects on finance, health, routine and leisure of the family, while the subjective burden was the perception of the adverse effects of illness. The course of the patient's disorder is influenced by the burden and the way families cope with it [8]. Family psychoeducational interventions have demonstrated reductions in family burden and reductions in the rate of illness relapse and severity of symptoms for the patients [9-12]. Alleviating burden and distress in caregivers has important economic and social benefits [13].

The addition of psychoeducation to pharmacological interventions brings benefits for the patient and the family [14-16]. The psychoeducational approach strives to empower family members to participate actively in the treatment of the patient [17].

In Pakistan, most patients with schizophrenia live in the community and are cared for in their homes by their family members. There are very limited community-based mental health services, halfway houses or alternative living facilities. The resources to support families are beginning to develop, but are very limited in the face of the huge demand. The reduction of family burden can help the families to sustain their caring role.

In the comprehensive Cochrane review of family interventions for schizophrenia that was updated in 2006, there was no study of family psychoeducational intervention from Pakistan [18]. In our literature search we could not identify any publications in this area from Pakistan.

In this paper, we report the results of a randomised controlled trial of the effects of psychoeducation on the family burden in Pakistan.

## Methods

This study had a between-group design and compared two sets of participants (patients and their family members). One group of family members received psychoeducation in addition to psychotropic drugs, and the other group received psychotropic drugs only. Both groups were assessed twice, prior to and 6 months after the psychoeducational intervention.

### Sample

The sample consisted of 108 patients of mixed sex and their family members; there were 52 in the group who received psychoeducation and 56 in the group who did not receive psychoeducation. The Diagnostic and Statistical Manual of Mental Disorders, fourth edition text revision (DSM-IV TR) diagnostic criteria were used for the selection of patients with schizophrenia.

Patients included in the study ranged in age between 18 and 45 years and had a history of two or more relapses during the course of their illness despite getting treatment. The patients and their families included in the study had homogenous sociodemographic characteristics. Patients manifesting schizophrenia-like symptoms due to any organic disorder such as dementia or any other cognitive impairment, abuse of alcohol or of drugs acting on the central nervous system and those with clinical evidence of epilepsy or intellectual disability were excluded.

One adult relative living with the patient in the same property, and who had maximum interaction with the patient or who was directly involved with the patient was included in the study. Mostly these were parents, spouses, siblings or any other significant relative. Family members with at least 5 years of school education were included so that they were able to understand and follow researchers' instructions and read the psychoeducation package.

### Assessment and intervention measures

At the time of first assessment a demographic information questionnaire was used to collect information regarding the demographic characteristics of the patient and the participating relative. The questionnaire was designed to collect information about age, sex, educational level, birth order, number of siblings, marital status, number of children, occupation and work status, and to gather details about the illness (age at onset, number of admissions in hospital, number of relapses, family history of mental illness and so on). The participant relatives' demographics included age, sex, education and relationship with the patient.

### Family Burden Interview Schedule (FBIS)

Pai and Kapur's Family Burden Interview Schedule [19] was used to assess family burden. The FBIS assesses the burden placed on families of psychiatric patients living in the community setting. This scale measures objective and subjective aspects of burden and it contains six general categories of burden, each having two to six individual items for further investigation. Subcategories include: financial burden, effects on family routine, effects on family leisure, effects on family interaction, effects on physical health of family members and effects on mental health of other family members. Each item is rated on a three-point scale, where 0 is no burden and 2 is severe burden.

### Psychoeducation intervention package

The psychoeducation package used in the current study was modelled after the psychosocial family intervention package used by Kuipers and Leff [20]. The psychoeducation booklet was translated into Urdu for the participants of the current study. The important components of the package are summarised below.

The intervention begins with providing information to family members about schizophrenia, and how it affects the persons' thoughts, emotions and behaviour. A detailed account of symptoms is provided. Disturbances in sensory perception and their effects on the behaviour of the patient are explained.

The family receives information about the possible causal factors. The family is informed about factors that influence the occurrence of schizophrenia, including genetics, neurochemistry, biological factors, life stressors and interpersonal and social factors.

The family and patient are educated about the treatment in detail. This component includes information about medication, its side effects and how these can be dealt with, likely benefits of the medicine, adherence to treatment, the importance of follow-up and information regarding prognosis.

The intervention emphasises the role the family can play in helping the patient to stay well. The intervention assists the families to improve their communication skills. Another important component of psychoeducation is to address the emotional upset in family members. A therapist helps family members to normalise the negative emotional responses by providing information regarding these issues.

One of the important aspects of the education package was to address family concerns and highlight their role in patient recovery and rehabilitation, which in turn will reduce the burden on family members. Family members were encouraged to address their own needs and to resume their former personal and social interests, which is imperative for their own mental health.

### Procedure

The project had ethical approval from University of the Punjab. Written informed consent from the participating patient and the accompanying family member was sought for participation in the research. A researcher personally assessed patients and relatives for suitability to participate in the study. The diagnosis for each patient was confirmed using DSM-IV TR criterion. The researcher was not involved in the randomisation of the patients and families, which was performed at the institution.

The participating family members were required to complete a family burden interview schedule before they participated in the intervention programme. The patients in the non-psychoeducation group, as well as their participant family members, were assessed using same tools at the same time.

Psychoeducation sessions were arranged in hospital settings. All the sessions were carried out individually with family and the patient at the Department of Psychiatry, Mayo Hospital, Lahore, Pakistan. Education sessions were based on a specially designed information booklet.

The first session aimed to provide general information about schizophrenia, its nature, types and causes. Common stereotypes were discussed and dispelled, providing accurate information about illness. In the second session, schizophrenia was explained as a syndrome affecting thoughts and emotions, which in turn results in disturbed behaviour. The distinction between positive and negative symptoms was explained so that relatives could understand the illness.

The third session focused on the importance of pharmacological treatment; information about side effects and the likely benefits of medication in acute and maintenance phase were described. The role of medication in relapse prevention was outlined. The important role of the family in recovery and rehabilitation of the patient was clarified. The fourth educational session provided general advice encouraging family members to address their personal and social needs to improve their well-being and to resume their formal social interests.

The family members of the patients who received psychoeducation attended a total of nine therapeutic sessions. Information about schizophrenia and its treatment was provided to family members in four sessions, held weekly. Sessions were interactive and participation was encouraged, which gave rise to further discussions. Families were encouraged to ask questions. In five follow-up sessions a monthly formal contact with the patient and participating relative was maintained.

In follow-up sessions, the clinical condition of the patient as reported by the family and patient was recorded. The family as well as the patient were invited to discuss any problems that they encountered during that period and any incidences of emotional upset, communication problems or relationship problems, and they were counselled accordingly. The average time for the two initial educational sessions was 1.5 h, and subsequent sessions were 30 to 40 minutes.

When the patient was in a stable phase a session was conducted with him/her individually, in which he/she was also educated regarding the nature and process of illness using the same educational pattern as was used for the family. However, while educating patients, the main emphasis was on compliance with pharmacological treatment and the likely benefits of taking medicine as well as advice for regular follow-up and to resume the normal day-to-day routine.

The patients in both groups were receiving equivalent doses of medication, but the psychoeducation group had the additional advantage of psychoeducational sessions.

The Urdu version of the psychoeducation booklet was distributed among participating relatives so that they could share knowledge with the remaining family members. The participating relatives in the two groups were requested to complete the FBIS after the completion of psychoeducation in the psychoeducation group.

### Statistical analysis

We analysed the data with SPSS V. 15 (SPSS, Chicago, IL, USA). The categorical variables were compared using the χ^2 ^test. We treated the FBIS scores as interval variables and used the t test to look at differences in the groups. For age and income we used the t test to compare groups. We utilised an intention to treat analysis for the analysis of family burden scores.

## Results

### Characteristics of the sample

A total sample of 108 patients were recruited and randomly assigned to the psychoeducation group (n = 52) or the non-psychoeducation group (n = 56). In all, 100 patients completed the treatment. One patient from the psychoeducation group had died by the 6-month follow-up period. Of the 108 patients, 58 (57.3%) were males and 50 (46.7%) were females. The mean age of the treatment group patients was 25.31 years (SD 7.02) and for control group was 27.00 years (SD 7.29). Table 1 gives the characteristics of the two groups.

**Table 1:** Demographic characteristics of the patients

Variable	Group		*P *values
		
	Experimental (n = 52)	Control (n = 56)	
Sex:			
Male, n (%)	26 (50%)	32 (57.1%)	0.56
Female, n (%)	26 (50%)	24 (42.9%)	
Mean (SD) age, years	25.31 (7.02)	27.00 (7.29)	0.22
Current work status, n (%):			
Working	5 (9.6%)	8 (14.3%)	0.45
Not working	47 (90.4%)	48 (85.7%)	
Education, n (%):			
Up to 5 years	5 (9.6%)	9 (16.1)	
5 to 10 years	28 (53.8%)	28 (50%)	
More than 10 years	19 (36.5%)	19 (33.9%)	
Mean (SD) family income in Pakistan Rupees	9,569 (5,032)	8,335 (5,983)	0.25
Marital status, n (%):			
Married	11 (21.2%)	12 (21.4%)	0.27
Unmarried	40 (76.9%)	39 (69.6%)	
Divorced	1 (1.9%)	5 (8.9%)	
Patient living with, n (%):			
Spouse	9 (17.3%)	8 (14.3%)	
Parents	42 (78.8%)	45 (80.4%)	0.86
Siblings	2	3 (5.4%)	

The two groups were similar and comparable in demographic characteristics. There were no significant differences between the two groups for age, gender, educational level, marital status or length of illness.

Most of the relatives were females (mothers, sisters and daughters). Relatives were living in the same household as the patient. The majority of the relatives had a primary level of education and about one-third had completed their education up to secondary school (Table 2).

**Table 2:** Demographic characteristics of the participant family members

Variable	Psychoeducation group (n = 52), n (%)	Non-psychoeducation group (n = 56), n (%)	*P *value
Sex:			0.54
Male	16 (30.8%)	21 (37.5%)	
Female	36 (69.2%)	35 (62.5%)	
Mean (SD) age of relative, years	41.84 (11.31)	44.25 (13.29)	0.31
Education:			0.51
Up to 5 years	15 (28.7%)	22 (39.3%)	
5 to 10 years	20 (38.5%)	18 (32.1%)	
Over 10 years	17 (32.7%)	16 (28.6%)	
Relationship with patient:			0.59
Father	8 (15.4%)	12 (21.4%)	
Mother	24 (46.2%)	25 (44.6%)	
Spouse	6 (11.5%)	2 (3.6%)	
Sister	6 (11.5%)	5 (8.9%)	
Brother	4 (7.7%)	7 (12.5%)	
Daughter	4 (7.7%)	5 (8.9%)	

To examine the efficacy of the psychoeducation intervention in reducing family burden in families of patients with schizophrenia, the subscale scores were derived according to the scoring method described by the authors. Scores for each scale were worked out by summing item scores. Owing to the varied number of items in different subscales, standard scores were computed. An independent samples t test was computed to compare the two groups on burden for pre-intervention and post-intervention scores. The results are given in Table 3.

**Table 3:** Independent samples t test analysis comparing two groups on pre-intervention family burden

Burden subgroup	Treatment group, mean (SD)	Control group, mean (SD)	t Value	*P *value
Financial	4.23 (2.13)	3.57 (2.13)	1.6	0.11
Routines	4.54 (1.60)	3.70 (1.91)	2.46	0.01
Leisure	4.25 (2.44)	4.11 (2.41)	0.30	0.76
Interaction	3.33 (2.10)	3.48 (2.15)	0.37	0.70
Physical health	2.60 (2.71)	2.11 (2.41)	0.99	0.32
Psychological health	2.38 (1.88)	2.07 (2.36)	0.75	0.45

Pre-intervention analysis revealed no differences in scores from the psychoeducation and non-psychoeducation group except for routine. Family members in the psychoeducation group reported a significantly greater burden for the 'routine' score as compared to the non-psychoeducation group.

To examine the differences in burden level between the two groups post intervention, an independent t test analysis was carried out. The results are displayed in Table 4.

**Table 4:** Independent samples t test comparing two groups post intervention on family burden

	Treatment group, mean (SD)	Control group, mean (SD)	t Value	*P *value
Financial	2.37 (1.6)	3.00 (1.78)	-1.93	0.056
Routines	1.46 (1.61)	2.38 (1.95)	-2.63	0.01
Leisure	1.77 (2.08)	3.46 (2.24)	-4.05	0.000
Interaction	1.33 (1.68)	2.80 (2.08)	-4.02	0.000
Physical health	1.29 (1.70)	1.57 (2.15)	-0.75	0.45
Psychological health	1.00 (1.35)	1.75 (2.16)	-2.13	0.035

There was a significant difference in post-intervention measures of burden in most categories. The groups were not different in the physical health category, and in financial category the result was just above 5% significance.

We finally compared the two groups while controlling for the pre-intervention scores. The results are shown in Table 5. The results show that if the baseline scores are controlled for, then the difference between the groups is significant for all categories of burden.

**Table 5:** Mean difference in scores between the groups

Subgroups of burden	T1	T2	T3	t Value	*P *value
Financial	0.65	-0.63	-0.43	6.65	0.00
Routines	0.84	-0.91	-0.38	4.20	0.00
Leisure	0.14	-1.69	-0.22	2.65	0.009
Interaction	-0.15	-1.47	-0.34	4.29	0.00
Physical health	0.48	-0.28	-0.34	5.21	0.00
Psychological health	0.31	-0.75	-0.36	4.86	0.000

T1 is the mean difference prior to intervention, T2 is the mean difference post intervention and T3 is the difference post intervention controlled for the pre-intervention scores.

The results showed a significant reduction in the level of burden in families who received psychoeducation as compared to those who did not receive psychoeducation. The psychoeducation group scored significantly lower on financial burden, social interaction and psychological health compared to the non-psychoeducation group. In the initial analysis both groups did not differ in burden in terms of leisure activities and effects on physical health. These differences became significant when the pre-intervention scores were taken into consideration.

## Discussion

The present study examined the role of psychoeducation in the alleviation of family burden. Patients with schizophrenia were randomly allocated to one of two groups, a psychoeducation group or a non-psychoeducation group. The findings of the present study showed that families receiving psychoeducation reported significantly lower burden compared to those who did not receive psychoeducation. Care for patients with schizophrenia at home constitutes a considerable burden on the family. Despite Pakistan having an extended family system, which is supportive in times of stress, the families of patients with schizophrenia were experiencing burden. It is clear that families feel an appreciable burden and find it difficult to cope with schizophrenia. They often lack knowledge about the nature of the patient's illness and receive little help from professionals for the management of the patients' behaviour. Coping with the patients' problems frequently results in adverse effects on physical and psychological health of the family members, so relatives should be provided with more information regarding illness and be given more support to alleviate the distress they feel [21].

Our findings are important because we have tested this intervention for the first time in Pakistan using measures that were developed in India, a country with a similar culture. Although there is plenty of literature from developed countries confirming the effectiveness of this approach [8], we thought it was important to test this method in Pakistan to see if it works in this setting.

In the current study, financial burden was high in both groups at time of preassessment. One could argue that the discontinuation or loss of job of the patient, as well as the difficulties faced by caring family members who may find it hard to continue work because of their extra responsibilities, would contribute to financial burden. Moreover, expenditures incurred on treatment, medicine, and at times transport due to hospitalisation away from home is also added reasons for financial burden on families. Our findings are in agreement with those of Rouget and Aubry [22], who in their study also reported financial burden in families of patients with schizophrenia. Bhagyalaxmi and Raval [23] also found (moderate to severe) financial burden in 86% of affected families.

The majority of the carers in current study, and other related studies, are women, so special attention needs to be paid to their needs in order to help them and share the responsibilities that these relatives have taken on. In the Pakistani context, the carers may benefit from support from members of the extended family to decrease the burden of care. Provision of respite care services is non-existent in Pakistan, even in big cities.

The development of an informal support network for patients and their families could reduce their isolation and burden and, in the majority of cases, should be a feasible option in Pakistan.

Psychoeducation should be offered to families as a matter of routine in Pakistan, and policymakers need to take these finding into account when planning services.

As financial burden is an important component of the total burden for the families, more local services provided either free or at a subsidised rate could reduce the burden.

## Competing interests

This was TN's PhD project.

## Authors' contributions

The project was jointly conceived and planned by both the authors. TN collected the data under supervision by RK. The paper was jointly written by both the authors.
